# Cognitive and Affective Correlates of Chinese Children’s Mathematical Word Problem Solving

**DOI:** 10.3389/fpsyg.2018.02357

**Published:** 2018-12-18

**Authors:** Juan Zhang, Sum Kwing Cheung, Chenggang Wu, Yaxuan Meng

**Affiliations:** ^1^Faculty of Education, University of Macau, Macau, China; ^2^Department of Early Childhood Education, The Education University of Hong Kong, Tai Po, Hong Kong

**Keywords:** word problems, executive function, math fact fluency, reading comprehension, math anxiety, reading anxiety, children, mathematics

## Abstract

Mathematical word problem solving (MWPS) involves multiple steps, including comprehending the problem statements, determining the arithmetic operations that have to be performed, and finding the answers. This study investigated the relative contributions of different cognitive and affective variables to children’s MWPS. To achieve this goal, 116 third-grade Chinese children were tested. Results showed that after controlling for age and non-verbal intelligence, the abilities to solve direct and indirect mathematical word problems were positively correlated with the working memory component of executive function, reading comprehension ability, math fact fluency and math anxiety. Moreover, math anxiety was found to fully mediate the relationships between reading anxiety and MWPS. Implications of the findings on how to promote children’s MWPS skills were discussed.

## Introduction

One of the major goals of mathematics learning is to know how to apply mathematical concepts to solve problems in everyday life (Kilpatrick et al., 2001). Some children, however, struggle with mathematical word problem solving (MWPS) (Hegarty et al., 1995). This happens perhaps because MWPS is not a simple task but involves at least three steps. Children have to represent the problem situation, choose a solution strategy, and apply the strategy to obtain the answer (Willis and Fuson, 1988). Therefore, MWPS does not only call for children’s mathematical knowledge, but also their general cognitive skills (such as the abilities to focus only on relevant information in the problem statements, storing information of the problem situation in the working memory while retrieving possible solution strategies from the long-term memory) as well as their reading skills (Stern, 1993). Occasionally, the situation is complicated by the fact that some children possess high levels of mathematics or reading anxiety, and such negative feelings may greatly affect their performance (Tsui and Mazzocco, 2006; Wu et al., 2012; Piccolo et al., 2017; Sorvo et al., 2017). In view of the above, the present study was interested in investigating the relative contributions of different cognitive and affective variables to children’s MWPS.

### Word Problems: A Combination of Reading Comprehension and Mathematical Problems

Mathematical word problems refer to mathematical problems that are embedded in story contexts. Children are thus required to integrate their linguistic and basic calculation skills to find out their solutions (Ostad, 1998). One of the many ways to classify mathematical word problems is based on the level of consistency between the language used in the story and the arithmetic operation that are called for. In direct problems, the arithmetic operation required is consistent with the relational term used in the problem (e.g., performing “addition” for a problem with the relational term “more than”) (Lewis and Mayer, 1987; Parmar et al., 1996). In contrast, in indirect problems, the arithmetic operation required is inconsistent with the relational term used in the problem (e.g., performing “addition” for a problem with the relational term “less than”) (Lewis and Mayer, 1987; Parmar et al., 1996). The present study sought to examine the correlates of children’s performance on these two types of problems because we would like to know whether the language used in mathematical word problems would affect the types of cognitive skills that were required to solve them, and the results might inform how to help those who were weak in solving different types word problems.

Several past studies have demonstrated the significant role of language and literacy skills in MWPS. Lee et al. (2009) found that children’s reading comprehension ability accounted for a significant portion of the variance in their representation of algebraic word problems. In the study of Hegarty et al. (1995), compared to unsuccessful problem solvers, successful problem solvers were less likely to adopt a direct translation strategy, i.e., simply looking for cues from numbers and keywords to come up with a plan of solving the problem. Instead, they tended to comprehend the problem and transform the problem statements into a mental representation of the problem situation (Hegarty et al., 1995). This perhaps suggests that MWPS, to certain extent, requires a deep level of reading comprehension.

On the other hand, MWPS is, no surprise, a good indicator of mathematical proficiency. In the study of Jitendra et al. (2005), children’s performance in their word problem-solving measures was positively correlated with their mathematical concepts and computational skills. Lee et al. (2009) also showed that children’s ability to discern quantitative relationships was a positive correlate of their ability to represent algebraic word problems. Interestingly, Fuchs et al. (2006) found that children’s fluency in retrieving addition and subtraction facts, but not their algorithmic computation ability, predicted their performance in arithmetic word problems.

### Anxiety and Task Performance

Anxiety can impair cognitive functioning. As sub-types of anxiety, reading anxiety and math anxiety are not exceptions and are found to be linked with individuals’ performance in respective domains. Zbornik and Wallbrown (1991) found that there was a negative correlation between reading anxiety and reading achievement among upper primary school students. Similarly, Kuşdemir and Katrancı (2016) showed that fourth graders’ higher levels of reading anxiety were associated with poorer performance in a reading comprehension test. Based on a review of findings from the Program for International Student Assessment (PISA) and a number of experimental studies, Foley et al. (2017) concluded that math anxiety was negatively correlated with math performance. The relationship was evident across countries and was likely bidirectional in nature (Foley et al., 2017). Carey et al. (2016) also supported the view of bidirectionality. As they noted, laboratory studies suggested that experimentally induced anxiety could lower individuals’ performance on math tasks, whereas data from children with dyscalculia and longitudinal studies revealed that poor math performance could evoke math anxiety (Carey et al., 2016).

To account for the mechanisms of how anxiety hampers cognitive performance, Eysenck et al. (2007)’s attentional control theory suggested that anxiety might make the individual less capable of inhibiting incorrect responses and more susceptible to distraction (e.g., threat-related stimuli that are irrelevant to the task demands, worrying thoughts). Moreover, anxiety might reduce the individual’s ability to switch attention between tasks and process the secondary task in dual-task situations (Eysenck et al., 2007).

Despite the fact that correct comprehension of the problem statements was the very first step for successful MWPS, no existing studies have examined the relative roles of reading anxiety and math anxiety in MWPS. The present study thus seeks to fill in this research gap. As demonstrated in past studies, the nature and effects of reading anxiety and math anxiety seemed to be intertwined with each other. Carey et al. (2017) found that the higher the level of math anxiety of primary and secondary school students in their sample, the poorer their mathematics as well as reading performance. In the study of Punaro and Reeve (2012), 9-year-old children who reported high levels of worries toward the language task also found the mathematical task worrying, whereas those who reported high levels of worries toward the mathematical task regarded the language task as less worrying than the mathematical task. Punaro and Reeve (2012) then concluded that literacy anxiety might be a maniefestation of general academic anxiety but math anxiety was more domain-specific. Based on this theoretical notion, we speculate that math anxiety (which is a type of anxiety specifically related to the task under investigation) would mediate the relationship between reading anxiety (which is a sign of general academic anxiety) and MWPS.

### Executive Function and Mathematical Learning

Executive function can be defined as the abilities to control and shift attention in a flexible manner, inhibit impulsive responses and retain information in working memory (Blair, 2016; Cantin et al., 2016). These abilities set the foundation for us to make plans, regulate emotions and control the display of impulsive acts (Blair, 2016). Different researchers have used different ways to measure executive function. Two common methods include behavioral rating scales (which can be self-rated, or rated by others like parents and teachers) and performance-based measures (e.g., Color Word Stroop task, Dimensional Change Card Sorting task) (Toplak et al., 2013; Cantin et al., 2016).

Numerous past studies have shown that children’s executive function was correlated with mathematical proficiency. Blair and Razza (2007) found that after controlling for non-verbal intelligence, young children’s inhibitory control (one component of executive function) was positively associated with their early mathematical ability. Bull and Scerif (2001) found that after controlling for intelligence and reading ability, in addition to inhibition efficiency, children’s mathematical ability was also related to their perseveration and working memory span. Cantin et al. (2016) found that there was a direct positive linkage between elementary school children’s cognitive flexibility and mathematical ability. Working memory, inhibitory control and cognitive flexibility also had indirect contributions to mathematical ability through reading comprehension ability (Cantin et al., 2016). In the study of Cragg et al. (2017), working memory, on the one had, had a direct positive association with attainment in mathematics. On the other hand, the two variables were indirectly associated via knowledge of number facts, knowledge of conceptual principles underlying arithmetics and skills in performing arithmetic procedures (Cragg et al., 2017). Individuals’ inhibitory control as demonstrataed in a numerical task also had indirect linkages with mathematical achievement via knowledge of number facts and skills in performing arithmetic procedures (Cragg et al., 2017).

As suggested by Cawley and Miller (1986), in order to solve mathematical word problems, children have to analyze the problem situation by selecting useful information from the problem statements, followed by determining which strategy can best help solve the problem. In light of this, executive function is expected to play an important role in MWPS. For instance, Blair et al. (2015) found that there were robust associations between young children’s exeuctive function and MWPS. Swanson et al. (2008) revealed that children’s accuracy in MWPS could be predicted by two components of working memory (namely central executive and visual-spatial sketchpad), as well as the growth of two components of working memory (i.e., central executive and phonological storage). Lee et al. (2009) showed that children’s working memory contributed to their problem representation and solution formation for algebraic word problems. Fuchs et al. (2006) found that after controlling for non-verbal reasoning, children’s attention level (as indicated by teachers’ rating of their inattentive behaviors) was a positive correlate of their arithmetic word problem solving. However, working memory did not uniquely explained variance in arithmetic word problem solving, as it lost its explanatory power when phonological decoding and sight word efficiency were included in the path analysis model. Best et al. (2011) further found that children’s executive function had a stronger correlation with MWPS than calculation abilities. They speculated that it was because calculation might just require the individual to retrieve mathematics facts from long-term memory, whereas MWPS often involves more plan generation and higher levels of self-monitoring (Best et al., 2011).

Even more, with emotional control as one of its components, executive function may help children regulate the negative emotions, such as anxiety, induced during the learning process. Jain and Dowson (2009), for example, found that there was a negative correlation between children’s self-regulation and mathematics anxiety. Bradley et al. (2010) found that after receiving training on emotion self-regulation, high school students in their sample showed lower levels of test anxiety. Lyons and Beilock (2012) observed the brain activities of university students with high levels of math anxiety when they were attempting a mathematical task. Based on their findings, they concluded that those who could control their cognitive resources (such as shifting attention and inhibiting predominant responses) before the task and reappraise negative emotional responses during the task tended to perform better (Lyons and Beilock, 2012). Despite the above, no existing studies have considered the contributions of math anxiety and reading anxiety when examining the relationships of executive function and domain-specific variables to MWPS. Moreover, the role of executive function in different types of MWPS has minimally been investigated. Compared to direct MWPS, we speculate that indirect MWPS require more executive function resources to solve, because children have to inhibit their intuitive response of carrying out arithmetic operations simply based on the relational term given (Daroczy et al., 2015). They also have to be more cognitively flexible in order to rephrase the inconsistent relational sentence and represent the problems properly (Lewis and Mayer, 1987).

### Present Study

As discussed, MWPS is a crucial part of mathematics learning. Despite the attention received by various researchers, the contributions of different cognitive skills (including general and domain-specific ones) to children’s MWPS have seldom been compared, and the potential role of affective variables in children’s MWPS has often been overlooked. The present study thus sought to examine Chinese children’s MWPS in relation to an array of cognitive and affective variables.

The cognitive variables under investigation included: (1) non-verbal intelligence, (2) executive function (including five components, namely inhibit, shift, emotional control, working memory, and plan/organize), (3) math fact fluency, and (4) reading comprehension. The first two were selected because these general cognitive skills play an important role in many types of cognitive processing (Fuchs et al., 2006; Jain and Dowson, 2009; Lee et al., 2009). The third was selected because good foundation skills in mathematics might facilitate children to solve higher-level mathematical problems (Binder, 1996; McCallum et al., 2006). The last was selected because past studies have found a close linkage between children’s literacy and mathematical development (Purpura et al., 2011), and MWPS required children to represent the problem embedded in a piece of text mathematically (Hegarty et al., 1995). Meanwhile, the affective variables of interest were: (1) math anxiety and (2) reading anxiety. These two variables are selected because it is not uncommon for children to have these kinds of anxiety, and these negative feelings have often been to hinder children’s performance in related tasks (Piccolo et al., 2017; Sorvo et al., 2017).

To obtain a more comprehensive capture of children’s MWPS skills, their performance was assessed with two tasks, namely direct and indirect problems. Compared to direct problems, indirect problems may be more challenging. This is because children have to be careful not to misinterpret the relational statements and perform arithmetic operations that are inconsistent with the relational term used in the problem statements (Lewis and Mayer, 1987; Daroczy et al., 2015). The whole indirect MWPS process may thus call for more and a wider range of cognitive resources.

Behavioral rating scales would be used to measure executive function, math anxiety and reading anxiety, whereas performance-based measures would be adopted to assess the remaining variables. We rely on behavioral rating scale rather than performance-based measure for executive function because two of the variables under focus were related to anxiety. We thus wanted to assess executive function skills as displayed in natural everyday life rather than stressful test situation. The behavioral rating scale could allow us to assess the “emotional control” component of executive function.

Based on results of past studies (e.g., Fuchs et al., 2006; Lee et al., 2009; Punaro and Reeve, 2012; Cantin et al., 2016; Carey et al., 2017), the followings are hypothesized:

H1a: Children’s MWPS is positively related to their non-verbal intelligence, executive function, math fact fluency, and reading comprehension ability.
H1b: Children’s MWPS is negatively related to their math anxiety and reading anxiety.
H2: Compared to direct MWPS, indirect MWPS has stronger correlations with executive function and reading comprehension ability.
H3: Children’s math anxiety mediated the relationship between their reading anxiety and MWPS.

To test our hypotheses, six-step hierarchical linear regression analyses would be performed on direct and indirect MWPS, respectively. In the first step, age and non-verbal IQ would be entered, so as to control their effects on direct and indirect MWPS. In the second step, executive function would be entered because this could allow us to understand the extent to which the contribution of the domain-general variable (i.e., executive function) to MWPS was shared by the domain-specific ones. Instead of combining the five components of executive function into one latent variable and entering it into the regression equation, the five components would be entered in stepwise fashion. This was important because indirect MWPS might require higher levels of inhibition of prepotent response than direct MWPS, and such analytic approach could help us know whether different component(s) of executive function had differential associations with direct and indirect MWPS. In the third step, math fact fluency would be entered because it assessed one’s basic mathematical abilities and was apparently the most relevant to MWPS. Adding it into the regression equations at this stage could let us examine at later stages whether other domain-specific cognitive and affective variables could make unique contributions to MWPS after controlling one’s basic mathematical abilities. The remaining three variables (i.e., math anxiety, reading anxiety, and reading comprehension ability) would be entered alternatively in the fourth to sixth steps. This allowed us to investigate whether each of them could make unique contributions to children’s MWPS. To test Hypothesis 3, Baron and Kenny (1986) procedures would be used, as it has extensively been used for mediation analysis among past studies, including recently published ones (e.g., Zhang et al., 2017; Kam et al., 2018; Mazzone et al., 2018).

## Materials and Methods

### Participants and Procedure

The participants were 116 third-grade primary students (61 boys and 55 girls, mean age = 9.60 years, *SD* = 0.50) from Zhuhai, Guangdong Province of China. All participants were typically developing children and written consent forms were collected from their parents or guardians prior to the formal tests. The experimental procedures were approved by the Ethics Committee of University of Macau. All tests were carried out in accordance with the approved guidelines and regulations. Six tasks were administered to each child through two sessions, including non-verbal intelligence, reading comprehension, reading anxiety (session 1), executive function, math fact fluency, MWPS, and math anxiety (session 2). The interval between the two sessions was about 1 week and each session lasted for about 2 h. Children could ask for a rest during the assessment. The executive function of children was evaluated by their parents using the BRIEF scale.

### Measures

#### Non-verbal Intelligence

Non-verbal intelligence was assessed using the Raven’s Progressive Matrix (Set A and Set B) (Kratzmeier and Horn, 1980). Specifically, two sets were used with each contained 12 items. During the assessment, the child was tested independently by research assistants and was asked to choose one piece of figure that could best fit the missing part of a visual geometric picture from six options. Each correct answer was scored as 1 and the maximum total score was 24 (Cronbach’s α = 0.79).

#### Executive Function

The Behavior Rating Inventory of Executive Function (BRIEF) (Gioia et al., 2000) was used, because it was a standardized questionnaire and had widely been used to assess the executive function (EF) of children aged from 5 to 18 years old among past studies (e.g., Anderson, 2002; Isquith et al., 2004; Roth et al., 2014). There were two versions of the BRIEF scale (teacher version and parent version). In the present study, only the parent version was adopted because parents were likely to be more familiar with children’s performance in everyday life. Five dimensions of children’s EF, including inhibit (10 items), emotional control (10 items), shift (8 items), working memory (10 items) and plan/organize (12 items), were assessed. For each item, parents were asked to rate on a three-point scale. The higher the children were scored, the worse their EF was (Cronbach’s α = 0.83 for inhibit, Cronbach’s α = 0.73 for shift, Cronbach’s α = 0.73 for emotional control, Cronbach’s α = 0.85 for working memory, and Cronbach’s α = 0.78 for plan/organization).

#### Reading Comprehension

During the task (Zhang et al., 2014), children were asked to read each of the given sentences silently and then choose one picture that best fits the sentence in meaning. There were 30 items, and their difficulty levels were appropriate for third-grade primary children in China. The maximum score was 30 (Cronbach’s α = 0.67).

#### Math Fact Fluency

This test was adopted and modified from the tasks used by Fuchs et al. (2006) and Nosworthy et al. (2013). In this test, 50 addition, 50 subtraction, and 50 multiplication items were presented to students, respectively, and students were asked to finish answering each type of items in 1 min. Before the test, the test paper was sent out to students with the back of the paper upward and no student was permitted to answer the questions until the research assistant told them to start. All answers should be provided within 1 min and students should stop answering when the time was over. Each correct answer was scored as 1 and the maximum score for the test was 150 (Cronbach’s α = 0.89).

#### Math Word Problem Solving

With reference to related past studies (Lewis and Mayer, 1987; Nesher and Hershkovitz, 1994; Parmar et al., 1996), 22 two-step mathematical word problems were created in accordance with the mathematical abilities and social experience of third-grade students in China. Of the two steps, one step involved addition or subtraction, and the other step involved multiplication or division. Half of the problems were direct problems, in which the relational term used in the problem was consistent with the arithmetic operation required for one step of the problem (e.g., A diary book costs $23. Uncle Ho bought 72 diary books. Uncle Wong bought 25 diary books fewer than Uncle Ho. How much should Uncle Wong pay?). The remaining half was indirect problems, in which the relational term used in the problem was inconsistent with the arithmetic operation required for one step of the problem (e.g., An exercise book costs $23. Uncle Yeung bought 72 exercise books. Uncle Yeung bought 28 exercise books more than Uncle Lee. How much should Uncle Lee pay?). The presentation order of the two kinds of problems was counterbalanced among participants, and participants were asked to solve all the problems within a limited period of time. The maximum score for each problem type was 11 (Cronbach’s α = 0.81 for direct problems and 0.74 for indirect problems).

#### Math Anxiety

The Test Anxiety Scale of Pintrich et al. (1991) was adopted. The original items about general test anxiety were modified to make them specific to mathematics (e.g., changing the word “test” to the phrase “math test”). Five 7-point items, ranging from 1 (totally incorrect) to 7 (totally correct), were used to evaluate the math anxiety. The participants were asked to choose the number of degree that best fit their status toward math. A sample item was “I have an uneasy, upset feeling when I take a math exam.” The maximum score for the scale was 35 (Cronbach’s α = 0.76).

#### Reading Anxiety

This scale consisted of five items. The items and instructions given to participants were exactly the same as those of the math anxiety scale, except that the word “math” was replaced by the word “reading.” The maximum score was 35 (Cronbach’s α = 0.89).

## Results

The descriptive summary of all the variables is displayed in Table 1. As expected, the mean score of indirect MWPS (3.11) was significantly lower than that of direct MWPS (3.78), *t*(115) = 4.81, *p* < 0.001, showing that indirect word problems were harder than direct word problems.

**Table 1:** Descriptive statistics for all measurements.

	Minimum	Maximum	Mean	*SD*
Non-verbal IQ	12.00	24.00	20.99	2.56
EF – Inhibit	37.00	69.00	49.96	7.94
EF – Shift	36.00	74.00	50.32	9.24
EF – Emotional control	36.00	67.00	48.92	8.20
EF – Working memory	38.00	74.00	52.68	8.24
EF – Plan/Organize	33.00	58.00	51.66	5.33
Math fact fluency	42.00	150.00	97.37	24.19
Direct MWPS	0.00	9.00	3.78	2.42
Indirect MWPS	0.00	10.00	3.11	2.20
Math anxiety	1.00	7.00	2.82	1.68
Reading comprehension	4.00	28.00	20.99	4.53
Reading anxiety	1.00	7.00	3.07	1.74

*The scores of the EF dimensions had been transformed to standard scores according to the BRIEF Manual.*

Table 2 shows the correlations and partial correlations (with age and non-verbal IQ controlled) among all the variables. As illustrated in Table 2, the construct validity of executive function measurement was confirmed by the strong associations among the four dimensions (i.e., inhibit, shift, emotion control, and working memory), except the “plan/organize” dimension. Both the zero-order correlation and partial correlation analyses suggested that two aspects of executive function (i.e., inhibit and working memory) strongly correlated with direct and indirect MWPS. As expected, math fact fluency and math anxiety were significantly correlated with both direct and indirect MWPS. Reading comprehension and reading anxiety were also strongly associated with direct and indirect MWPS.

**Table 2:** Correlations and partial correlations among variables.

	1	2	3	4	5	6	7	8	9	10	11	12	13
1. Age	1.00												
2. Non-verbal IQ	−0.10	1.00											
3. EF – Inhibit	−0.01	−0.09	1.00	0.60^∗∗∗^	0.64^∗∗∗^	0.74^∗∗∗^	0.01	−0.16	−0.26^∗∗^	−0.24^∗∗^	0.12	−0.13	0.21^∗^
4. EF – Shift	−0.18	−0.10	0.60^∗∗∗^	1.00	0.70^∗∗∗^	0.59^∗∗∗^	0.08	−0.17	−0.13	−0.18	0.14	−0.09	0.17
5. EF – Emotional control	−0.13	0.08	0.62^∗∗∗^	0.69^∗∗∗^	1.00	0.59^∗∗∗^	0.02	−0.07	−0.09	−0.09	0.05	−0.12	0.09
6. EF – Working memory	0.10	−0.07	0.74^∗∗∗^	0.60^∗∗∗^	0.59^∗∗∗^	1.00	0.07	−0.13	−0.31^∗∗^	−0.29^∗∗^	0.07	−0.17	0.15
7. EF – Plan/Organize	−0.51^∗∗^	−0.07	0.02	0.17	0.07	0.12	1.00	0.16	−0.04	−0.08	−0.08	0.04	0.07
8. Math fact fluency	−0.31^∗∗^	0.05	−0.15	−0.10	−0.03	−0.10	−0.28^∗∗^	1.00	0.46^∗∗∗^	0.44^∗∗∗^	−0.15	0.18	−0.08
9. Direct MWPS	−0.12	0.12	−0.27^∗∗^	−0.12	−0.07	−0.30^∗∗^	−0.01	0.47^∗∗^	1.00	0.79^∗∗∗^	−0.40^∗∗∗^	0.35^∗∗∗^	−0.30^∗∗^
10. Indirect MWPS	−0.22^∗^	0.19^∗^	−0.25^∗∗^	−0.15	−0.04	−0.27^∗∗^	−0.03	0.48^∗∗^	0.80^∗∗^	1.00	−0.39^∗∗∗^	0.33^∗∗∗^	−0.32^∗∗^
11. Math anxiety	0.08	−0.13	0.130	0.14	0.04	0.07	−0.09	−0.17	−0.41^∗∗^	−0.41^∗∗^	1.00	−0.18	0.77^∗∗∗^
12. Reading comprehension	−0.08	0.21^∗^	−0.15	−0.09	−0.09	−0.17	0.06	0.20^∗^	0.36^∗∗^	0.36^∗∗^	−0.20^∗^	1.00	−0.16
13. Reading anxiety	−0.02	−0.10	0.21^∗^	0.18	0.08	0.16	0.09	−0.08	−0.30^∗∗^	−0.32^∗∗^	0.77^∗∗^	−0.17^∗^	1.00

*Correlations among variables are presented below the diagonal; partial correlations controlling for age and non-verbal intelligence are presented above the diagonal. ^∗^p < 0.05; ^∗∗^ p < 0.01; ^∗∗∗^ p < 0.001.*

To further examine the contributions of executive function, math fact fluency, reading anxiety, math anxiety, and reading comprehension to direct MWPS and indirect MWPS, six-step hierarchical regressions were conducted (see Tables 3, 4). As described earlier, age and non-verbal IQ were entered as control variables in the first step. In the second step, the five components of executive function were entered by stepwise. Only working memory remained in the model and accounted another 9% variance for direct MWPS and 8% variance for indirect MWPS. Math fact fluency was entered in the third step and could account for another 17% variance for direct MWPS and 15% variance for indirect MWPS. Reading anxiety could account for extra 5% variance for direct MWPS and 6% variance for indirect MWPS when age, non-verbal IQ, working memory, and math fact fluency were statistically controlled. In addition, math anxiety could uniquely predict both direct (5%) and indirect (3%) MWPS in step 5. More importantly, reading comprehension could still account for 3% variance for both direct and indirect MWPS in step 6, indicating a unique role of reading comprehension in solving math word problems. We performed another set of hierarchical regressions to investigate the role of reading comprehension in the relationship between reading anxiety and MWPS. Results showed that reading anxiety could still account for 4% variance for direct and indirect MWPS respectively after reading comprehension was controlled. Finally, with a purpose to explore the role of math anxiety in the relationship between reading anxiety and MWPS, another set of hierarchical regressions was conducted. Surprisingly, after math anxiety was entered in step 5, reading anxiety could not predict direct or indirect MWPS in step 6, suggesting that the relationship between reading anxiety and MWPS was possibly mediated by math anxiety.

**Table 3:** Hierarchical regression predicting direct math word problem solving.

Step	*β*	*T*	*R*^2^	*ΔR*^2^
1 Age	−0.11	−1.16	0.03	0.03
Non-verbal IQ	0.11	1.19		
2 EF – Working memory	−0.31	−3.419^∗∗^	0.12	0.09^∗∗^
3 Math fact fluency	0.44	5.22^∗∗∗^	0.29	0.17^∗∗∗^
4 Reading anxiety	−0.23	−2.92^∗∗^	0.34	0.05^∗∗^
5 Math anxiety	−0.37	−3.07^∗∗^	0.39	0.05^∗∗^
6 Reading comprehension	0.19	2.49^∗^	0.42	0.03^∗^
4 Reading comprehension	0.24	2.95^∗∗^	0.34	0.05^∗∗^
5 Reading anxiety	−0.20	−2.63^∗^	0.38	0.04^∗^
6 Math anxiety	−0.34	−2.91^∗^	0.43	0.05^∗∗^
4 Reading comprehension	0.24	2.95^∗∗^	0.34	0.05^∗∗^
5 Math anxiety	−0.30	−3.97^∗∗∗^	0.42	0.08^∗∗^
6 Reading anxiety	0.06	0.50	0.42	0

*^∗^p < 0.05; ^∗∗^ p < 0.01; ^∗∗∗^p < 0.001.*

**Table 4:** Hierarchical regression predicting indirect math word problem solving.

Step	*β*	*T*	*R*^2^	*ΔR*^2^
1 Age	−0.21	−2.27^∗^	0.08	0.08^∗^
Non-verbal IQ	0.17	1.86		
2 EF – Working memory	−0.28	−3.24^∗∗^	0.16	0.08^∗∗^
3 Math fact fluency	0.42	4.99^∗∗∗^	0.31	0.15^∗∗∗^
4 Reading anxiety	−0.25	−3.23^∗∗^	0.37	0.06^∗∗^
5 Math anxiety	−0.29	−2.44^∗∗^	0.40	0.03^∗^
6 Reading comprehension	0.18	2.26^∗^	0.43	0.03^∗^
4 Reading comprehension	0.22	2.73^∗∗^	0.36	0.05^∗∗^
5 Reading anxiety	−0.23	−2.96^∗∗^	0.40	0.04^∗∗^
6 Math anxiety	−0.27	−2.29^∗^	0.43	0.03^∗^
4 Reading comprehension	0.22	2.73^∗∗^	0.36	0.05^∗∗^
5 Math anxiety	−0.28	−3.80^∗∗∗^	0.43	0.07^∗∗∗^
6 Reading anxiety	−0.02	−0.17	0.43	0

*^∗^p < 0.05; ^∗∗^ p < 0.01; ^∗∗∗^ p < 0.001.*

**FIGURE 1:**
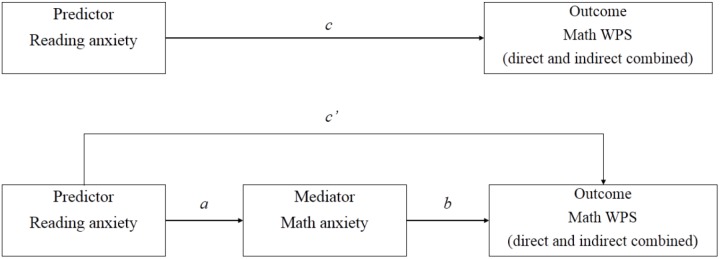
Math anxiety mediating reading anxiety and math WPS.

To provide more direct evidence confirming that reading anxiety was associated with direct MWPS and indirect MWPS with the mediation of math anxiety, we combined direct MWPS and indirect MWPS as math word problem solving (WPS) and conducted additional hierarchical regression analysis. Four conditions should be met to confirm the full mediation effect (Baron and Kenny, 1986). First, the reading anxiety (predictor) should be significantly regressed (*c*) on math WPS (outcome). Second, the mediator (math anxiety) should also be associated with the outcome (*b*). Third, the predictor and mediator should be closely related (*a*). Fourth, the predictor should be non-significant after mediator was controlled (*c’ < c*). As illustrated in Table 5, reading anxiety (condition 1) and math anxiety (condition 2) could both predict math WPS (direct and indirect MWPS combined), respectively. In addition, reading anxiety was significantly associated with math anxiety (condition 3). More importantly, reading anxiety failed to predict math WPS after math anxiety was statistically controlled. Therefore, the result showed that reading anxiety was fully mediated by math anxiety when predicting math WPS (see Table 5 and Figure 1).

**Table 5:** Hierarchical regression examining the mediating effect of math anxiety.

	Condition 1		Condition 2		Condition 3		Condition 4	
	*β*	*t*	*β*	*t*	*β*	*t*	*β*	*T*
Reading anxiety	−0.33	−3.69^∗∗∗^	–	–	0.77	12.94^∗∗∗^	0.02	0.12
Math anxiety	–	–	−0.43	−5.11^∗∗∗^			−0.44	−3.33^∗∗∗^

*^∗^p < 0.05; ^∗∗^p < 0.01; ^∗∗∗^p < 0.001.*

## Discussion

The present study examined the relative contributions of different cognitive and affective variables to children’s MWPS. Our findings suggested that after controlling for age and non-verbal IQ, children’s MWPS (no matter for direct or indirect problems) was only significantly correlated with the working memory component of executive function, math fact fluency, reading comprehension and math anxiety. Regarding the relationships of executive function and reading comprehension to MWPS, their strengths were similar across direct and indirect problems. Moreover, the association between reading anxiety and MWPS was fully mediated by math anxiety.

Partially different from our initial speculations, only two of the five components of executive function (i.e., inhibit and working memory) had significant but weak zero-order correlations with MWPS (including direct and indirect problems). No significant associations were found for the remaining three components (i.e., shift, emotional control, and plan/organize). However, when age, non-verbal intelligence and the five components were considered together in the regression equations, only working memory remained as a significant correlate. This is somehow similar to the findings by Lee et al. (2009), in which only working memory, but not inhibition and mental flexibility, were significantly related to skills of solving algebraic problems. Moreover, different from our hypothesis, the strength of the relationship between executive function and MWPS did not vary much across direct and indirect problems. These findings perhaps suggest that working memory shares some overlapping roles with other components in MWPS but its role is relatively more prominent. This happens possibly because during MWPS, children have to handle multiple tasks within a fairly short period of time, such as comprehending the problem statements, memorizing various pieces of useful information about the problem situation, forming a mental representation of the problem situation in mathematical terms, recalling possible solution strategies (Willis and Fuson, 1988). With better working memory, children can perform the aforesaid tasks more efficiently (Lee et al., 2009). Meanwhile, compared to working memory, other components of executive function might be relatively less crucial, given that the mathematical word problems involved were not very complicated in nature (i.e., two-step word problems only).

Consistent with our initial speculation and results of past studies (Fuchs et al., 2006), math fact fluency was a significant correlate of children’s direct and indirect MWPS. To recall, the math fact fluency task required children to retrieve basic addition, subtraction and multiplication facts accurately and quickly. The ability to perform such a task well might thus help children to free up more mental resources to handle more complicated tasks required in the process of MWPS (Wong, 1986).

As expected, reading comprehension was a significant correlate of both types of MWPS. This again shows that it is important for children to have a deep comprehension of the problem statements, instead of just relying on the keywords in the problem statements (e.g., the number words, the relational terms) to solve the mathematical word problems (Hegarty et al., 1995). However, different from our hypothesis, the strength of its relationship with MWPS did not differ much across direct and indirect problems. This happens perhaps because inconsistent language only appeared in one of the several problem statements in indirect problems. Thus, compared to direct problems, solving indirect problems might not call for much extra reading comprehension skills.

Of the two affective variables under investigation, math anxiety seemed to be more relevant to MWPS than reading anxiety was. On the one hand, though reading anxiety had significant zero-order correlations with both types of MWPS, it was not a significant correlate when math anxiety was included in the regression equations. On the other hand, even after controlling for all the cognitive variables, math anxiety could still account for additional variance in both types of MWPS. These results, somehow, are not surprising for at least two reasons. First, as proposed by Punaro and Reeve (2012), reading anxiety can be regarded as a sign of general academic anxiety. It may therefore lose its power to explain variations in children’s MWPS when it is considered together with math anxiety (i.e., a specific type of anxiety associated with the academic domain under examination). Second, reading comprehension is only the very first step of MWPS, and literal understanding the problem situation does not necessarily lead to a correct answer. The fear of failure in representing the problem situation from a mathematical perspective and performing the required arithmetic operation might thus be more closely related to the outcome of MWPS, i.e., obtaining the correct answer. Indeed, high level of math anxiety can hinder children’s performance on mathematical tasks by creating a disruption of their working memory (Ashcraft and Kirk, 2001). Meanwhile, it is also possible that children with high level of math anxiety might be less willing to engage in math-related tasks in their everyday life, which in turn make them have fewer opportunities to practice their math skills and develop competency in MWPS (Fennema, 1989). Nevertheless, it should be noted that compared to the possible score range (i.e., 1–7), the mean scores of math anxiety and reading anxiety of our participants were only 2.82 and 3.07. In fact, in samples with higher levels of math anxiety or reading anxiety, the contributions of executive function and anxiety variables might become even more crucial, because individual might suffer from greater impairments on working memory and other executive function components (e.g., inhibit, plan/organize) and it might be more important to possess higher levels of the “emotional control” component for maintaining performance.

Findings of the present study can provide educators and parents with insights on how to promote children’s MWPS skills. First, when teachers observe that a child makes mistakes frequently when solving mathematical word problems, teachers have to examine more closely the reason(s) for such a situation. As shown in the present study, it might happen because the child shows difficulties in processing multiple pieces of information in the mind, comprehending the problem statements, and/or retrieving basic math facts to find out the answers. These different reasons indeed call for different approaches to help the child.

Second, the present study shows that teachers have to find out effective strategies to help children reduce math anxiety. This is because the fear induced by the necessity of tackling math problems might be so overwhelming that it can hinder children’s math performance, even though the children might have already possessed the required math knowledge and skills. Teachers and parents should thus talk to children who show high levels of anxiety, so as to understand the reasons underlying their anxiety and adopt corresponding strategies to relieve their stress.

The present study had several limitations that required attention. First, given that all variables were measured at one time point only, no causal relationships between the variables can be drawn. Future researchers can thus conduct longitudinal studies to examine the extent to which various cognitive and affective variables and their growth can predict children’s performance in MWPS in future.

Second, the present study only relied on parental report questionnaire to measure children’s executive function. Some recent studies (e.g., Toplak et al., 2008, 2013) have suggested that there were only modest correlations between the scores obtained from behavioral rating scales and performance-based measures and the two types of measures might assess two different cognitive levels. Performance-based measures might assess the efficiency of the cognitive processes that are employed for controlling behaviors, whereas behavioral rating scales might tap on the behaviors of how to achieve personal goals in real-life situations. In the future, researchers can therefore measure executive function using different methods and examined whether the patterns of results found in the present study still hold the same.

Third, the present study only focused on two types of word problems (i.e., direct and indirect two-step arithmetic word problems) and the outcome of children’s MWPS (i.e., the accuracy of their answers). In fact, the difficulty level of arithmetic word problems depends on a range of linguistic, numerical and contextual factors (Cummins et al., 1988; Davis-Dorsey et al., 1991; Daroczy et al., 2015), and there are different correlates of children’s performance in different cognitive phases of MWPS (Lee et al., 2009). Future studies can thus explore whether the variables associated with each MWPS stage are the same across problem types and test situations. This, in turn, can yield important implications on how to help children tackle different types of word problems.

In summary, the present study is one of the few studies to investigate the relative contributions of different cognitive and affective variables to children’s MWPS. Our findings showed that children’s MWPS was significantly correlated with their working memory, reading comprehension, math fact fluency and math anxiety. In order to provide more effective support to children struggling with MWPS, it is essential for teachers and parents to figure out in which aspect these children show difficulties and adopt corresponding strategies to help them overcome their barriers.

## Ethics Statement

This study was carried out in accordance with the recommendations of ethic guidelines, Institutional Review Board in the University of Macau with written informed consent from all subjects. The protocol was approved by the Institutional Review Board in the University of Macau.

## Author Contributions

JZ and SKC developed the research idea and research design. CW analyzed the data. JZ, SKC, CW, and YM wrote and reviewed the manuscript.

## Conflict of Interest Statement

The authors declare that the research was conducted in the absence of any commercial or financial relationships that could be construed as a potential conflict of interest.
